# Gallstone Disease and Microbiome

**DOI:** 10.3390/microorganisms8060835

**Published:** 2020-06-02

**Authors:** Irina N. Grigor’eva, Tatyana I. Romanova

**Affiliations:** Laboratory of Gastroenterology, Research Institute of Internal and Preventive Medicine-Branch of The Federal Research Center Institute of Cytology and Genetics of Siberian Branch of Russian Academy of Sciences, Novosibirsk 630089, Russia; igrigorieva@ngs.ru

**Keywords:** gallstone disease, microbiota, gut, bile acids, oral cavity, bile ducts, cholecystectomy

## Abstract

Gallstone disease (GSD) has, for many years, remained a high-cost, socially significant public health problem. Over the past decade, a number of studies have been carried out—both in humans and in animal models—confirming the role of the microbiota in various sections of the gastrointestinal tract as a new link in the etiopathogenesis of GSD. The microbiome of bile correlates with the bacterial composition of saliva, and the microbiome of the biliary tract has a high similarity with the microbiota of the duodenum. Pathogenic microflora of the oral cavity, through mechanisms of immunomodulation, can affect the motility of the gallbladder and the expression of mucin genes (*MUC1,* *Muc3, MUC4*), and represent one of the promoters of stone formation in the gallbladder. The presence of *H. pylori* infection contributes to the formation of gallstones and affects the occurrence of complications of GSD, including acute and chronic cholecystitis, cholangitis, pancreatitis. Intestinal bacteria (*Clostridium, Bifidobacterium, Peptostreptococcus, Bacteroides, Eubacterium*, and *Escherichia coli*) participating in the oxidation and epimerization of bile acids can disrupt enterohepatic circulation and lead to the formation of gallstones. At the same time, cholecystectomy due to GSD leads to the further transformation of the composition of the microbiota in various parts of the gastrointestinal tract, increasing the risk of developing stomach cancer and colorectal cancer. Further research is required to determine the possibility of using the evaluation of the composition of the microbiota of the gastrointestinal and biliary tracts as an early diagnostic marker of various gastroenterological diseases.

## 1. Introduction

Gallstone disease (GSD) is a frequent and socially significant public health problem worldwide [1]. Approximately 10–20% of adults in the United States have gallstones, and the prevalence of this disease is constantly growing. The number of cholecystectomies is also increasing and currently numbers more than 750,000 per year. Medical expenses for the prevention and treatment of cholelithiasis amount to $62 billion per year in the USA [2]. The cost of surgical care for patients with GSD in the United States is estimated to be $6.5 billion [3]. The European Association for the Study of the Liver (EASL) found that about 20% of Europeans have GSD [4]. In Germany, GSD occupies a leading position among the reasons for hospital admissions of patients with gastroenterological conditions [5]. More than 175,000 cholecystectomies are performed annually because of cholelithiasis [5]. Despite the high worldwide prevalence of GSD, the role of the biliary microbiota in gallstone pathogenesis remains obscure. In 1966, Maki [6] showed the role of bacterial infection in the pathogenesis of pigmented gallstones. Later, in several works, it was demonstrated that changes in the gut microbiota are also one of the etiological factors of cholesterol gallstones [7,8,9].

There is currently an increasing number of studies about the role of the gut microbiome as a key link in the pathogenesis of GSD [10,11,12,13,14,15,16]. The main factors contributing to the formation of gallstones are defective gallbladder motility, metabolism and secretion of cholesterol and bile acids [12]; the gut microbiota is actively involved in the regulation of bile acid metabolism, changing the size and composition of the bile acid pool [13,14]. These associations are being verified by a significantly increasing number of studies confirming the participation of intestinal microbiota in gallstone formation, including works studying the bacterial metabolome and antibiotic resistance genes [10]. In addition, some types of intestinal bacteria can elicit chronic inflammation and reactive oxygen species (ROS)-mediated genotoxicity or secrete DNA-damaging toxins, which also increases the formation of gallstones [17,18,19].

## 2. Oral Cavity Bacterial Communities and GSD

The oral cavity is the gateway to the digestive and respiratory systems, and it is highly vascularized, resulting in potential implications of the oral microbiome in other systemic diseases: cardiovascular [20] and endocrine [21,22] diseases, cancer [23,24,25], rheumatoid arthritis [25,26], etc. To date, a fairly large amount of research has been accumulated proving the connection of diseases of the gastrointestinal tract with the oral microbiome [27].

The total surface area of the oral cavity is about 214.7 ± 12.9 cm^2^ [28]. The oral microbiome is a unique and extensive ecosystem—including viruses, fungi, protozoa, archaea, and bacteria [29,30]—with over 700 prokaryote species [28] that interact in various ways to form biofilms. Individuals’ oral microbiomes are highly specific at the species level, although the human oral microbiome shows few geographical differences overall [30]; however, the number of microbes of different types can fluctuate. In the oral cavity, 50–200 types of microorganisms belonging to 15 genera are isolated, which are found in almost every person [29,31]. The microbiome of a healthy oral cavity contains five predominant types: 96% of the total number of taxa belong to *Firmicutes* (genus *Streptococcus*, family *Veillonellaceae*, genus *Granulicatella*), Proteobacteria (genus *Neisseria*, *Haemophilus*), *Actinobacteria* (genus *Corynebacterium*, *Rothia*, *Actinomyces*), *Bacteroidetes* (genus *Prevotella*, *Capnocytophaga, Porphyromonas*), *Fusobacteria* (genus *Fusobacterium*) [31], and *Spirocheetes* [32]; the remaining types, *Euryarchaeota*, *Chlamydia*, *Chloroflexi*, SR1, *Synergistetes*, *Tenericutes*, and *TM7* represent the remaining 4% of taxa [32]. It should be noted that there is a constant flow of organisms entering the oral cavity from the environment, which differs from endogenous species; i.e., local microbes. It is believed that all genera from *Bacteroidetes* are related to the host, while almost all genera from *Alphaproteobacteria* are transients of the environment [32]. The quantitative composition of the microbiota can be influenced not only by changing environmental conditions, but also by age, diseases, medications taken, etc. [33].

At the same time, the bile microbiome correlates to a great degree with the bacterial composition of saliva, and the biliary tract microbiome has a relatively higher similarity with the duodenal microbiota [34]. Shen et al. [35] identified 13 novel biliary bacteria based on whole-metagenome shotgun sequencing (WMS): 8 of the 13 novel species were human oral microbial taxa; the rest were of possible environmental taxa origin.

Oral bacteria can directly or indirectly modulate the microbiome of the gall bladder and upper gastrointestinal tract, participating in the pathogenesis of GSD. Oral bacteria disrupt the synthesis of NO, the cofactor of eNOS, both in the vascular network and in the colon, and reduce the expression of the antioxidant protein Nrf2 and the bioavailability of NO, increasing the amount of reactive oxygen species [36].

Oral bacteria have been implicated in gallstone pathogenesis, although a clear understanding of the mechanisms of their influence on the cholelithigenesis is lacking. In a population study conducted in the United States (the Third National Health and Nutritional Examination Survey 1988–1994—NHANES III), including 995 adults with GSD and 10232 controls aged 20–74 years, a univariate analysis found that predictors of GSD are poor oral hygiene (odds ratio (OR) = 1.7, 95% confidence interval (CI) 1.1–1.25, *p* = 0.02) and missing teeth (OR = 4.8, 95% CI 3.1–7.4, *p* < 0.001), and multivariate analysis confirmed that missing teeth are an independent predictor of GSD (adjusted OR = 1.7, 95% CI 1.1–2.8, *p* = 0.02) [37]. In the bile of patients with GSD, the most common inhabitants of the human digestive tract are *Proteobacteria, Firmicutes, Bacteroidetes, Actinobacteria, Fusobacteria*, as well as *Synergistetes* and TM7 [34]. The *Pyramidobacter* genus, which belongs to the phylum *Synergistetes*, has been isolated mainly from the human oral cavity. *Pyramidobacter* and three *Enterobacteriaceae* genera (*Escherichia*, *Klebsiella*, and an unclassified genus) were highly abundant in the majority of bile samples. The dominant biliary genera in patient with GSD (e.g., *Prevotella*, *Rothia*, and *Haemophilus*) were relatively more aligned with the patient’s salivary microbiota [34].

Oxidative stress is thought to play an important role in the pathophysiology of GSD: GSD patients have a high level of oxidative stress in the gall bladder mucosa, which might result in an altered gall bladder absorption and secretion of bile components such as mucins and glycoproteins. The resultant increased risk of bile supersaturation would further contribute to the progress of gall stone formation [38].

The transcription nuclear factor-erythroid (NF-E) 2-related factor 2 (Nrf2), not only as an antioxidant but also as a regulator of the transcription of a wide array of genes involved in drug metabolism and detoxification, may also be important in protecting against gallstone development [39]. Transcription factor (*Nrf2*) maps to the Lith 1 loci. Hepatic *Nrf2* gene and protein expression is also increased in gallstone-resistant AKR/J strain compared with gallstone-susceptible C57L/J strain mice, identifying *Nrf2* as a putative *Lith 1* gallstone gene [40].

The oral microbiota has been considered to be a biomarker for metabolic syndrome [41] and cardiovascular diseases [42]; that is, those diseases that are closely related to GSD [16,43]. The validation of the identified oral bacteria by quantitative polymerase chain reaction (PCR) showed that healthy controls possessed significantly lower levels of *G*. *adiacens* (*p* = 0.023) and a higher ratio of *Peptococcus* to *Granulicatella* (*p* < 0.05) than metabolic syndrome subjects [41]. The authors support the idea that local oral microbiota and these microbial biomarkers can be associated with systemic disorders. Teles et al. (2012) investigated the correlation between oral parameters of inflammation and the levels of systemic biomarkers [42]. They concluded that “the quality and quantity of the host response to oral bacteria may be an exposure more relevant to systemic atherothrombotic coronary events than clinical measures”: the presence of serum antibodies to *P. gingivalis* increased the risk of stroke (1.6–2.3 times), while periodontal diseases are associated with elevated systemic levels of high-sensitivity plasma C-reactive protein (CRP). In addition to CRP, elevated systemic levels of interleukin (IL)-6 have been reported, a major inducer of the acute phase reaction, as well as higher levels of fibrinogen and IL-18 in the plasma of periodontitis subjects. An elevated risk for atherosclerosis is correlated with increases in CRP, fibrinogen, and pro-inflammatory cytokine levels. These findings suggest a role for oral bacterial species, as potential sources of systemic inflammatory biomarkers, particularly periodontal pathogens, in atherogenesis [42]. Chhibber-Goel et al. (2016) confirmed the presence of 23 oral commensal bacteria, either individually or in co-existence, within atherosclerotic plaques in patients undergoing endarterectomy. Of these 23 bacteria, five (*Campylobacter rectus*, *Porphyromonas gingivalis*, *Porphyromonas endodontalis*, *Prevotella intermedia*, *Prevotella nigrescens*) are unique to coronary plaques [20]. The relationship between atherosclerosis and all components of the metabolic syndrome and GSD is well known [16,43].

Congenital immunostimulation, including from the pathogenic microflora of the oral cavity, affects the secretion of cholecystokinin [44], the main factor involved in the emptying and filling of the gallbladder [19]. A microbiome changes the expression of mucin genes (*MUC1, Muc3,* and *MUC4* genes) through immunomodulation, thereby changing the accumulation of mucin gel, which is the nucleation matrix for the formation of cholesterol gallstones in the gallbladder [8,19].

This involvement of the oral microbiome in various mechanisms of GSD development has led to research aimed at studying it as an object for the primary and secondary prevention of GSD.

## 3. Biliary Tract Microbiome

It was previously suggested that a healthy biliary system is sterile [45]; however, several years ago, it was recognized that the gallbladder has a complex microbiota in non-pathological conditions. There are a number of possible routes of migration and colonization of the biliary tract by bacteria: for example, translocation from the duodenum through the Oddi sphincter or hematogenous entry into the liver with further excretion into the bile [46,47]. Previous research has linked biliary infection with gallstone development and indicated that bacteria may act as the nucleating factor initiating the formation of both pigment and cholesterol gallstones [7,48,49]. Results of the culture method established the coexistence of biofilm-forming bacteria in bile and gallbladder/gallstones (*Pseudomonas aeruginosa, E. coli, Klebsiella pneumoniae, Enterococcus* species (spp.), and *Acinetobacter* spp.) [50,51,52] in different combinations, the presence of *Capnocytophaga* spp., *Lactococcus* spp., *Bacillus* spp., *Staphylococcus haemolyticus*, *Enterobacter* or *Citrobacter* spp., *Morganella* spp., *Salmonella* spp., and *Helicobacter pylori* (*H. pylori*) was also characterized in these samples by the polymerase chain reaction-denaturing gradient gel electrophoresis (PCR-DGGE) method [52,53]. This infection was detected in 54.5 % (24/44) of patients with gallbladder stones [52].

## 4. *Helicobacter Pylori* and *Helicobacter* spp. in Bile, Gallbladder Tissue, and Gallstones

Many studies have shown that *H. pylori* and enterohepatic strains of *Helicobacter* contribute to the formation of cholesterol gallstones [54,55,56,57,58,59,60,61,62,63,64]. *H. pylori* is a Gram-negative, spiral-shaped, motile microorganism. Since the discovery of *H. pylori*, a number of additional *Helicobacter* spp. has been isolated from the stomachs and intestinal tracts of a variety of mammalian species; From 18 to 25 separate *Helicobacter* spp. have been recognized [65].

Certain species of *Helicobacter*, including *H. bilis* and *H. hepaticus*, inhabit the intestine and invade the bile ducts and liver [47,66,67]. Due to its sensitivity to bile [67,68,69], *H. pylori* was not detected in bile samples [61,69,70] or gallbladder mucosa [68] in patients with biliary tract diseases, and it was demonstrated that unconjugated bile salts were more toxic than the conjugated salts [69].

*Helicobacter* spp. and *Helicobacter pylori* in bile samples, gallbladder tissue, and gallstones were detected by several methods: using cultures [52,59,67,71], by immunohistochemistry [46], by urease test, Giemsa, and immunohistochemical stain [72], by 16S rRNA PCR-amplification, by nested and multiplex PCR [47,54,55,56,59,60,61,62,63,64,67,71,73,74,75,76,77], by PCR-DGGE [52], both by PCR and by an ELISA study of the specific antigen (H. pylori stool antigen) [47], by ELISA after a preabsorption step using *H. pylori* antigens [46,78], by a Western blot analysis of *Helicobacter* antibodies [67], or by WMS sequencing and 16S rRNA sequencing on bile samples [35].

One reason for the conflicting results regarding the presence of *H. pylori* in biliary pathways is the use of different methods for its detection by different authors. Another reason, which could be important in establishing an association between *Helicobacter* infection and gallstones, is that different samples have been tested in different studies: bile, stone, and tissue from patients with gallstones have been considered.

After cholecystectomy, the gallbladder mucosa from 94 patients with symptomatic GSD was investigated in terms of *H. pylori* by urease test, Giemsa, and immunohistochemical stain; the study demonstrates the presence of *H. pylori* in the gallbladders of 37% (from any of the three tests) of patients with symptomatic GSD [72]. A study from Greece indicated positive *Helicobacter pylori* serology in 51.3% of patients with calcular biliary and pancreatic disease [79]. However, there is cross-reactivity among *H. bilis, H. hepaticus*, and *H. pylori* [78].

In order to identify *Helicobacter* in gallstones of Iranian patients with biliary disease, gallstone and bile samples from 33 patients were subjected to rapid urease test, culture and Multiplex PCR [71]. In 18.1% of stone and 12.1% of bile samples from 33 patients, *H. pylori* DNA was detected using Multiplex PCR. Rapid urease and culture tests were negative for all samples. The PCR was negative in the control group [71].

Cultures of 102 bile samples from patients with biliary diseases, including 74 patients with GSD, for *Helicobacter* spp. did not show any growth, but the presence of *Helicobacter pylori* DNA by PCR was detected in 3.92% of them. No significant association was found between the development of the diseases and presence of the bacteria [75].

In the case-control study from Iran, 77 patients with (n = 52) and without (n = 25) gallstones were included [59]. Bile culture samples were all negative for *Helicobacter* species. *H. pylori* is very difficult to grow on culture media because of the microaerophilic characteristics of this organism as it dies if it has any contact with air. The PCR technique has a high sensitivity and specificity compared to routine bacterial culture [71].

*Helicobacter* spp. DNA were detected in the bile of 19.2% of patients with gallstone cholecystitis; of these, eight (15.3%) were *H. pylori* and two (3.9%) were *H. bilis*. No *Helicobacter* species were detected in gallstones by PCR [59]. Fatemi et al. found an association between the presence of *H. pylori* DNA in the bile and acute gallstone cholecystitis [59]. There is no statistically significant correlation between three enterohepatic *Helicobacter* spp. (*H. bilis, H. hepaticus*, and *H. pullorum*) and GSD [59]. However, patients with GSD (41%) had a significantly higher (*p* =0.029) prevalence of *Helicobacter hepaticus* infection in bile than samples from patients with other diseases. The authors conclude that *Helicobacter hepaticus* may be closely associated with diseases of the liver and biliary tract in humans [67]. Antibodies to *H. hepaticus* were detected more frequently in patients with GSD [80].

Silva et al. [55], in a clinical study of gallbladder and bile tissue from 46 Brazilian subjects with and 18 without GSD, showed a direct and independent relationship between GSD and the presence of *Helicobacter* DNA (detected by a nested PCR assay) in gallbladder tissue (*p* = 0.009; OR = 14.72; 95% CI = 1.97 to 108.90). The sequences of the 16S rRNA genes were >99% similar to that of *Helicobacter pylori*. These results support the hypothesis that *Helicobacter* is associated with the pathogenesis of human GSD [55].

*Helicobacter*-produced urease was reported to promote calcium precipitation, which might initiate gallstone formation [57]. *H. pylori* infection affects the pathophysiology of gallbladder stone formation and its complications, including cholecystitis, cholangitis, pancreatitis, and biliary cancer [59]. One mechanism is the release of large amounts of proinflammatory and vasoactive substances, such as interleukins IL-1, IL-6, and tumor necrosis factor (TNF)-α, which are involved in gallbladder inflammatory disorders and the pathogenesis of GSD [81]. In addition, producing oxidative stress and free radical reactions in the gallbladder wall and bile can induce gallstone formation [82].

The CagA protein of *H. pylori* has been found to have a homology with aminopeptidase and can therefore increase the risk for gallstone formation [49]. *H. pylori* may also promote the risk of stone formation by acting as a foreign body to form a nidus around which the stone may develop, or it may produce hydrolyzing enzymes or nucleating proteins such as immunoglobulins [75]. However, Griniatsos et al. [83], in contrast, consider that *H. pylori* does not have a lithogenic potential for the formation of cholesterol gallstones. Stathopoulos et al. [84] suggest that *H. pylori* infection may affect gallbladder function, namely leading to a decrease in concentration ability.

A retrospective study performed in Beijing (7803 subjects) revealed that the prevalence of gallstones in the group of *H. pylori* (+) individuals was 1.53 times lower than in the group of *H. pylori* (-) (*p* = 0.012) [58]. Another study showed the absence of a clear link between GSD and the colonization of human gallbladder tissue by *Helicobacter* in the Mexican population [76].

The presence of *H. pylori* infection also affects the occurrence of complications of GSD, including acute and chronic cholecystitis, cholangitis, and pancreatitis. In a study in Iran, a relationship was found between the presence of *H. pylori* DNA and acute stone cholecystitis [59], although a recently published meta-analysis confirmed a direct association of *H. pylori* infection with an increased risk of chronic cholecystitis and GSD: OR = 3.022 (95% CI, 1.897-4.815; I^2^ = 20.1%) [85]. Moreover, the odds ratios (95% CI) for research in the Asian and non-Asian regions were slightly different and amounted to 3.75 (1.83–7.71) and 2.25 (1.29–3.89), respectively [85]. These data demonstrate that there may be racial and demographic differences in the etiology of gallstones.

The degree of colonization of *H. pylori* in the gastric mucosa decreased after cholecystectomy from 69.6% to 41.3% of patients (*p* < 0.0001) [86]. In Japanese and Chinese studies, it was proved that the presence of *H. pylori* is positively associated with gallstones, and the eradication of *H. pylori* can even lead to the prevention of gallstones [87,88].

Although Figura et al. [89] suggested 20 years ago that *H. pylori* being present in human bile samples may be a factor that increases the risk of gallstones, the role of *Helicobacter* in the pathogenesis of human GSD is still unclear. Further studies are needed to determine if *Helicobacter* spp. is a cofactor of, or a cause of, biliary tract disease.

It has been noted that biliary stones are frequently encountered in areas endemic for opisthorchiasis: the presence of parasite eggs was demonstrated in the stones [90]. Saltykova et al. [91] demonstrated that infection of fish-transmitted *Opisthorchis felineus* modified the biliary microbiome. The difference in the bile bacteria communities between patients with GSD infected with *Opisthorchis felineus* and uninfected at the taxonomic levels from type to genus was significant, although unstable. At the genus level, 22 phylotypes differed between these two groups, including the presence of species of the genera *Mycoplana*, *Cellulosimicrobium*, *Microlunatus*, *Phycicoccus*, *Archaeans genus*, and *Halogeometricum,* and increased numbers of *Selenomonas, Bacteroides, Rothia, Leptotrichia, Lactobacillus, Treponema*, and *Klebsiella*, although the alpha diversity of the biliary tract microbiome does not appear to be affected by infection with *Opisthorchis felineus* [91].

## 5. Bile Acids, Microbiota, and Bile Lithogenicity

The main components of bile are bile acids, at about 50%; additionally, cholesterol and fatty acids (about 20%), phospholipids, bilirubin, etc. are present [92,93]. According to Wang et al. [8], the interactions of five primary defects play a critical role in the pathogenesis of cholesterol GSD: genetic factors and Lith genes; the hypersecretion of cholesterol in the liver, leading to oversaturated gallbladder cholesterol; fast phase transitions due to the accelerated crystallization of cholesterol and the growth of crystals of solid cholesterol; impaired gallbladder motility; and intestinal factors, including an increased amount of absorbed cholesterol being delivered from the small intestine to the liver for the hypersecretion of the biliary tract, as well as changes in the gut microbiota. Secondary hydrophobic deoxycholate bile salt may be one of the possible links between bile lithogenicity and gut motility: patients with GSD have a longer transit through the colon, more common and Gram-positive anaerobes, and more pronounced 7α-dehydroxylating activity in the cecum than normal subjects [94].

Bile acids play an important role in digestion by emulsifying and solubilizing fats. Disorders of bile acid metabolism are the leading factors in the pathogenesis of cholesterol GSD [95]. Moreover, in recent years, the amount of accumulated data of preclinical and clinical studies has increased sharply, which indicates a significant role of the metabolic activity of biliary and gut microbiota in the pathogenesis of GSD [96]. *Firmicutes, Proteobacteria,* and *Bacterioidetes* dominate the human bile microbiome in patients with GSD [12]. In almost all stages of bile formation, the microbiota of the gastrointestinal and biliary tracts are involved, including the regulation of lipid metabolism, cholesterol metabolism, biotransformation, and enterohepatic circulation of bile acids.

The liver synthesizes two primary bile acids from cholesterol, cholic acid (CA) and chenodeoxycholic acid (CDCA), which are conjugated to either taurine or glycine before being poured into the bile flow. Conjugated bile acids are the primary components of bile, which are deconjugated by intestinal bacteria. The bile acid profile excreted in feces, mainly composed of secondary BAs, largely depends on the gut microbiota metabolism, but bile acids are emerging as regulators of the gut microbiome at the highest taxonomic levels [17].

The microbiota is involved in the regulation of hydrolysis of bile acids to constituent components, cleavage of exogenous aromatic rings, deconjugation of bile acid complexes by hydrolytic enzymes, and the formation of free bile acids [15]. The main stages of the biotransformation of bile salts during their enterohepatic circulation include the deconjugation of conjugated bile acids by bile salt hydrolase (BSH) by optional and anaerobic bacteria in the small intestine; the 7α-dehydroxylation of CA and CDCA to form deoxycholic acid (DCA) and lithocholic acid (LCA), respectively, which makes bile salts available as substrates for further modifications of gut microbiota [80]; and the 7β-dehydroxylation of bile acids by ursodeoxycholic acid (UDCA) with the formation of LCA [97].

The gut microbiota-mediated biotransformation of the bile acid pool regulates bile acids signaling by affecting the activation of host bile acids receptors such as nuclear receptor farnesoid X receptor (FXR), which governs bile, glucose and lipid metabolism [15]. Indeed, a disrupted gut microbiota including reduced bile metabolizing bacteria significantly impairs bile acids metabolism and consequently the host metabolic pathways regulated by bile acid signaling, affecting glucose and cholesterol homeostasis [18], which is very important for cholelithogenesis [16]. Bile acid nuclear receptors are expressed in such tissues as heart and adipose tissue, the condition of which plays a significant role in the development of GSD [17]. In addition to affecting bile metabolism within the gut, the microbiota might also contribute to GSD pathogenesis through other mechanisms including increased energy intake, intestinal permeability, and contribution to chronic pro-inflammatory states [15]. A dynamic equilibrium exists between the diet–gut microbiome–bile acid pool size/composition, and perturbations in this equilibrium can result in pathological states (GI cancers, gallstones, etc.) [17].

Each factor that disrupts enterohepatic circulation leads to the formation of gallstones [80]. An increased level of secondary bile acids correlates with an increased risk of GSD. Secondary bile acids (DCA and LCA) are involved in the pathogenesis of cholesterol GSD [98]. In patients with GSD, the level of 7α-dehydroxylating bacteria was more than 42 times higher than in patients without GSD (*p* < 0.01) [99].

The antibiotic treatment of patients with cholesterol gallstones (a group with a high content of DCA) significantly reduced the levels of fecal 7a-dehydroxylating bacteria, DCA in bile, and the index of cholesterol saturation in bile [98]. The oxidation and epimerization of 3-, 7-, and 12-hydroxy groups of bile acids in the gastrointestinal tract is carried out using hydroxy steroid bile acid dehydrogenase (HSDH), which is expressed by many gut bacteria (e.g., *Clostridium, Bifidobacterium, Peptostreptococcus, Bacteroides, *Eubacterium**, and *Escherichia coli*) [100]. However, the Cys-1 mutation in serine or threonine cancels BSH activity in *Bifidobacterium bifidum* [101]. Only a small number of bacterial species belonging to the class *Clostridia* possess 7α/β-dehydroxylation activity [102]; for example, thanks to *Clostridium absonum*, which has both 7α and 7β-HSDH, toxic CDCA turns into a less toxic hydrophilic UDCA, which increases the chances of bacteria surviving in a highly competitive environment in the gut lumen [97].

By changing the composition of bile acids, gut bacteria can alter the cellular metabolism and physiology of the host and the metabolism of cells exposed to bile acids. In total, 63 gut microbes of a person were revealed to be associated with activity to lower cholesterol [103]: the first was *Bacteroides sp.* strain D8 of human origin [104]. *Bifidobacteria* have been proven to lower cholesterol in bile by assimilation or precipitation. Thus, when cultivating *Bifidobacterium breve* and *Lactobacillus amilovorus* in a nutrient medium with bile or taurocholic acid, it was found that the metabolites of *Lactobacilli* contribute to lowering cholesterol, activating its precipitation, and *Bifidobacteria* activate both the assimilation and precipitation of cholesterol [105,106]. On this basis, the conclusion was reached that the removal of cholesterol from the culture medium of *Bifidobacteria* and *Lactobacilli* is the result of the deconjugation of its bile salts and is not associated with the absorption of cholesterol.

A certain gut microflora produces cholesterol reductase, which catalyzes the conversion of cholesterol to insoluble coprostanol, which is subsequently excreted in the feces, thereby also reducing the amount of exogenous cholesterol [107]. A meta-analysis showed that probiotics (*L. acidophilus*, *B. lactis*, *VSL # 3*, and the *L. plantarum group*) can significantly reduce total serum cholesterol [105]. The study proved that capsule probiotics provide a new, non-pharmacological alternative for the prevention and treatment of lipid-associated diseases. The consumption of a BSH-positive strain of *Lactobacillus* significantly reduced cholesterol in patients with hypercholesterolemia [108]. Despite some positive results, the real contribution of these microbial groups to lowering cholesterol and molecular activity remains largely unknown.

The biliary tract microbiota is actively involved in mechanisms that ensure the prevention of colonization of the biliary tract by exogenous microorganisms and their immunological tolerance. Bile acids are ligands of farnesoid X nuclear receptor (FXR), which is involved in maintaining blood cholesterol homeostasis by regulating bile acid synthesis and the transport of bile salts and controls dyslipidemia, which is a risk factor for GSD [109]. FXR agonists, by affecting the amount of solubilizing bile salts and phospholipids in bile, can prevent gallstones in this way [110]. FXR is activated by the primary bile acids, CA and CDCA, and the gut microbiota metabolizes these primary bile acids into secondary bile acids in the gut and thereby changes the activation and signaling of FXR [111]. Ways of influencing these signaling interactions of the ‘gut microbiota–bile acid–host’ system, from probiotics to dietary interventions, may become new strategies to manage diseases associated with impaired bile acid metabolism [112]. Gutiérrez-Díaz et al. [113] confirmed the association between diet, biliary microbiota and GSD: in patients with GSD, bile microorganisms were changed, dairy consumption was negatively related to the proportions of *Bacteroidaceae* and *Bacteroides*, and several types of fiber, phenolics, and fatty acids were associated with an abundance of *Bacteroidaceae*, *Chitinophagaceae, Propionibacteraceae, Bacteroides,* and *Escherichia‒Shigella*. Healthy diets and specific nutritional interventions, including increased fiber intake, probiotics and prebiotics, could lead to the restoration of beneficial bacteria and the diversity of microbiota, contributing to recovery [114].

Bile acids play a key role in preventing bacterial proliferation: they can have many effects on bacterial cells, including damaging membrane lipids, causing the abnormal coagulation and denaturation of intracellular proteins in bacteria and causing oxidative stress, as detergents can dissolve the bacterial membrane and damage bacterial DNA [66]. Inagaki et al. [115] proved the antibacterial effect of conjugated bile acids in the distal small intestine, taking into account the role of FXR in protecting the small intestine from bacterial invasion and bacterial translocation in general and in patients with impaired bile flow. The activation of FXR by conjugated bile acids induced the expression of genes whose products prevent the overgrowth of bacteria and promote epithelial integrity. In mice lacking FXR, bacterial growth in the ileum was increased and the epithelial barrier was broken.

Conjugated bile acids in the intestine are known to be toxic to bacteria, especially at low pH values, and it is believed that they affect bacterial growth in various areas of the gastrointestinal tract [116]. In addition, microorganisms must have good resistance to bile acids if they want to survive in the bile ducts and in the human gut [117]. The presence of BSH and some transport proteins increases the resistance of some microorganisms to bile salts [101] and facilitates colonization [118].

BSH may play a role in the regulation of intracellular pH in a bacterial cell and thereby contribute to resistance to bile acids at low pH [7]. Since BSH is associated with a greater ability to survive transit through the intestines, BSH activity is regularly included in the selection criteria for probiotics [118,119]. The ability to enzymatically hydrolyze bile salts was observed in 273 strains of *Bifidobacterium* and *Lactobacillus*, as well as in *Clostridium* spp., *Bacteroides* spp., *Enterococcus* [97], but it was absent in *L. Lactis*, *L. mesenteroides*, and *S. thermophilus* [120]. Moreover, it has been found that there are several genes that confer resistance to bile, including toxR, omp U, tolC, cmeABC, rlpB, yrbK, rpoS, damX, and gltK, for example, for *V. cholera, Campylobacter, Enterococcus faecium, Salmonella*, etc. [121]. Moreover, tolerance to bile salts is generally considered more important than other properties (tolerance to gastric and pancreatic juice) when selecting probiotic strains from *Bifidobacterium* [122]. Nevertheless, the effect of the bile environment, which is known to be usually hostile to most bacteria, on bile bacteria remains unclear [43].

Studies are being conducted with the aim of determining the possibility of using the evaluation of the composition of the microbiota of the gallbladder as an early diagnostic marker of gallstone formation [13].

## 6. Gallstone Microbiome

The presence of living bacteria in gallstones has been proven using electron microscopy, bacteriological cultivation, and molecular genetic methods [51,123,124,125].

In a study by Hazrah et al. [51], microorganisms were cultured from gallstone nuclei in 81% of cases of GSD and in 77% of cases of gallbladder cancer, regardless of the type and size of the stones. Bacteria were present in 75% of pigmented, 76% of mixed, and 20% of cholesterol stones [103].

According to Kose et al. [126], the composition of the intestinal microbiota can also influence the formation of the type of gallstones (cholesterol, pigment, mixed). Under the action of a deconjugating factor, β-glucuronidase produced by bacteria, the precipitation of calcium bilirubinate crystals occurs, which are conjugated with anionic glycoprotein, leading to the agglomeration of calcium bilirubinate crystals into macroscopic stones [6].

In studies by Stewart L. et al., the effects of bacterial factors on the composition and morphology of gallstones (beta-glucuronidase and phospholipase) and mucus, and their effect on the severity of infection were studied [50,127]. Two hundred and ninety-two patients were studied and 382 gallstones were cultured. The stones were examined using scanning electron microscopy and infrared spectrography. Bacteria were tested for the production of β-glucuronidase/phospholipase and quantitative production of mucus [50,127]. It has been proven that bacterial characteristics can control the formation of gallstones. There was more pigment in gallstones with bacteria producing phospholipases/glucuronidase (71% versus 26%, *p* < 0.0001), while mucus (or its absence) was associated (67%) with cholesterol stones (*p* < 0.031, all comparisons) [103]. Severe infections correlated directly with β-glucuronidase/phospholipase, creating a colonization surface (55% vs. 13% without, *p* <0.0001); however, regarding mucus formation (55% vs. 8%, mucus <75 or >75, *p* = 0.008) in the centers of cholesterol stones, bacteria that produce only mucus were most often determined [50].

In a study by Peng Y. et al. investigating the microbiome of gallstones and bile in patients with cholelithiasis, it was found that 30% of cultured strains from cholesterol gallstones secrete β-glucuronidase and phospholipase A2. In total, 14 genera of bacteria were identified in gallstones of cholesterol, and eight genera in bile. Pseudomonas spp. were the dominant bacteria in the gallstones of cholesterol and bile. The Pseudomonas aeruginosa strains had the highest β-glucuronidase activity and produced the highest concentration of phospholipase A2 [128].

There is another alternative mechanism for the formation of stones in the biliary tract: the formation of biofilms during the formation of pigment stones [129]. The agglomerating factor in this case is glycocalyx (anionic glycoprotein).

Differences in the functional metagenomes of microbial communities in pigmented and cholesterol gallstones were revealed [126]. Genes involved in biofilm formation were mainly extracted from *Klebsiella* and *Enterococcus* found in pigment stones, and bile resistance genes were present in *Escherichia*, *Shigella, Serratia*, *Bacillus*, and *Klebsiella* isolated from cholesterol stones. Furthermore, in the majority of cholesterol gallstones examined, Gram-positive bacteria prevailed that were not identified in pigment stones. In pigment stones, a high proportion of genes involved in carbohydrate metabolism was revealed, and in cholesterol stones, the profile in which protein metabolism prevailed was more active [126].

## 7. Gut Microbiome and Cholecystectomy

The composition of the gut and biliary tract microbiome varies significantly in patients with GSD and in healthy subjects [13,14,15]. In patients with GSD, the microbial diversity is reduced, accompanied by a decrease in the beneficial genus *Roseburia* [13], with an overgrowth of bacteria of the *Proteobacteria* type, including a wide range of pathogenic microorganisms, such as *Escherichia, Salmonella, Vibrio*, and *Helicobacter* [12]. In patients with GSD, the enrichment of *Anaerotruncus*, *Parabacteroides*, and *Paraprevotella* is noted over the age of 60 years, while in individuals without GSD, this increase was not detected [11].

Cholecystectomy leads to a significant change in the composition of the gut microbiota [9,12]. After cholecystectomy, an even more pronounced decrease in the actual number of taxa occurs compared with individuals without GSD [9,11] and an increase in the number of *Bifidobacterium* and *Anaerostipes Dorea* [11]. In some individuals, an increase in the species *B. obeum* and *V. Parvula* (type *Firmicutes*) [9,11] and *Bacteroidetes* [13] is noted.

In patients with cholecystectomy, the numbers of *Anaerotruncus, Parabacteroides*, and *Paraprevotella* are also significantly reduced, and there is no growth with age compared with individuals without GSD [11]. A decrease in the amount of *Bacteroides*, negatively associated with secondary bile acids, is probably one of the main reasons for the increase in the incidence of colorectal cancer in patients with cholecystectomy.

Changes in the microbiome that occur after cholecystectomy persist for a long time [11]. Such changes are probably mediated by an abnormal transintestinal flow of bile acids, which begin to act without the rhythmic function of the gallbladder, increase the loss of bile acids from the intestine [130] and change the gut immune homeostasis [131]. It has been proven that symptomatic gallstones and cholecystectomy are associated with an increased risk of developing stomach cancer [132,133], cancer of the small intestine [134], and colon cancer [23,131].

## 8. Conclusions

The development of biomarkers—considering the contribution of changes in the specific microbial metabolic activity regarding the composition of bile—which can predict the severity of the disease, its prognosis, and also the response to therapy without the need for a biopsy, is at the center of most modern genomic, transcriptome, proteomic, and metabolic studies. In the future, patients may be diagnosed and selected for treatment according to their molecular signatures.
